# Characterization of UGT71, a major glycosyltransferase family for triterpenoids, flavonoids and phytohormones-biosynthetic in plants

**DOI:** 10.48130/forres-0024-0032

**Published:** 2024-10-31

**Authors:** Yang Yang, Jia Wang, Fuchuan Han, Jiantao Zhang, Ming Gao, Yunxiao Zhao, Yicun Chen, Yangdong Wang

**Affiliations:** 1 State Key Laboratory of Tree Genetics and Breeding, Chinese Academy of Forestry, Beijing 100091, China; 2 Research Institute of Subtropical Forestry, Chinese Academy of Forestry, Hangzhou 311400, Zhejiang Province, China

**Keywords:** UGT71 family, Secondary metabolites, Glycosylation, Plant defence

## Abstract

UGT catalyzes the transfer of glycosyl molecules from donors to acceptors, and the glycosylation catalyzed by them is a modification reaction essential for plant cell growth, development, and metabolic homeostasis. Members of this class of enzymes are found in all areas of life and are involved in the biosynthesis of an extensive range of glycosides. This review aims to screen and collate relevant properties of the UGT71 family in plants and their functions in plant secondary metabolites. Firstly, we conducted a retrospective analysis of information about plant UGTs, before focusing on UGT71s through glycosylation of secondary metabolites (triterpenoids, flavonoids) and glycosylation of phytohormones (ABA, SA). Consequently, they play a pivotal role in plant defence, hormone regulation, and the biosynthesis of secondary metabolites, thereby enabling plants to adapt to changing environments. Further investigation revealed that UGTs (UGT71s) can enhance the adaptive and resistant potential of plants in the context of today's deteriorating growing conditions due to climate change impacts caused by global warming. Nevertheless, further in-depth studies on the intricate interactions among UGTs in plants are required to fully exploit the potential of UGTs in protecting plants against stress.

## Introduction

Plants have evolved to synthesize a vast array of secondary metabolites that are not essential for their primary growth and development but play a crucial role in their interaction with the environment, reproductive strategy, and defense mechanisms^[1]^. These metabolites can undergo various chemical modifications, such as hydroxylation, methylation, and glycosylation. Enzymes have evolved to introduce new functional groups to molecules, enabling this capability. Glycosylation reactions can occur on different functional groups including -OH, -COOH, -NH2, -SH, and C-C in a wide range of molecules such as proteins, carbohydrates, primary and specialized metabolites, and xenobiotics^[2]^. Plant secondary metabolites frequently possess carbon skeletons connected to one or more sugar moieties. The glycosylation of these metabolites plays a crucial role in influencing their solubility, biochemical characteristics, subcellular distribution, and biological function. This is because glycosylation alters the structure of the metabolites, making them more soluble and affecting their ability to interact with other molecules in the cell. Overall, glycosylation is an important process that plays a key role in the function of plant secondary metabolites^[3]^.

Glycosylation represents the last step in the biosynthesis of numerous natural compounds, and is a process that can occur during the final stages of synthesizing various molecules, including glycoproteins, proteoglycans, and hormones^[4]^. Cellular homeostasis is crucially maintained through the regulation of levels, activity, and localization of important cellular metabolites. This process is facilitated by glycosyltransferase enzymes (GTs), which play a key role in glycosylation. The products of glycosylation modifications encompass a range of compounds such as glycoproteins, glycolipids, and various small-molecule glycoside, or glycoester compounds, including anthocyanosides, flavonoid glycosides, glycosides or glycoesters of hormones, and similar compounds. Modifications by these enzymes, which occur in all living organisms, modulate the solubility, stability, bioavailability, and bioactivity of various small molecules^[5]^. Therefore, it is believed that glycosylation modification affects many aspects of plant growth and development and is an important mechanism for regulating cellular metabolic homeostasis^[2]^.

The enzyme that catalyzes plant glycosylation is uridine diphosphate glycosyltransferase (UDP-glycosyltransferase, UGT), which is a glycosyltransferase that utilizes uridine diphosphate (UDP)-activated sugar molecules as glycosyl donors. It is the largest glycosyltransferase superfamily in plants, with many glycosyltransferases related to the glycosylation of secondary metabolites and phytohormone glycosylation belonging to this superfamily^[6]^. It is widely accepted that global warming is responsible for an increase in the frequency, intensity and duration of climate extremes. Furthermore, socioeconomic exposure is considered to be a dominant factor in climate impacts^[7]^. Glycosylation is a crucial chemical process that regulates the production and role of various specialized metabolites essential for combating pathogens and environmental stresses. This indicates that UGTs have the potential to enhance plant resilience and survival under challenging growing conditions that may arise from climate change. In this study, the functional properties of subfamily UGT71 and the distribution characteristics of each family in different plant families are further summarized, based on a review of the structural properties and family classification of plant UGTs. This is to provide guidance for the discovery and identification of plant UGT71s genes, as well as for the study of sequence structure and catalytic function, and to promote the molecular breeding of plant UGT71s. Furthermore, members of the UGT71 family play a role in the homeostasis of triterpenoids, flavonoids (and their derivatives), and hormones. This contributes to the study of glycoside synthesis, which in turn protects the growth and development of plants.

## Glycosyltransferase, glycosylation and UDP glycosyltransferase

**Figure 1:**
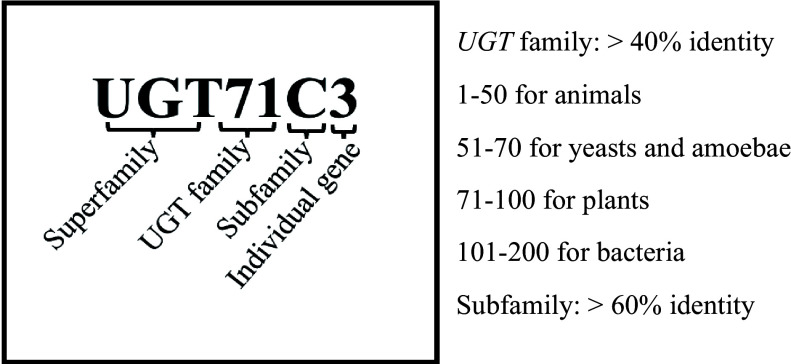
The nomenclature system for UGT genes.

Glycosyltransferases (GTs, EC 2.4.x.y) are a diverse family of genes found in various living organisms^[4]^. Glycosyltransferases (GTs) are enzymes that transfer sugar molecules from activated donors to specific receptors. The classification of UDP-glucuronosyltransferase (UGT) genes into 106 families (GT1~GT106) is based on substrate specificity, amino acid sequence similarity, and catalytic specificity. In Fig. 1, the nomenclature of UGT genes follows a pattern where the root symbol UGT is followed by an Arabic numeral representing the family, a letter indicating the subfamily, and another Arabic numeral for the individual gene. The numbering system assigns families 1−50 to animals, 51−70 to yeast, 71−100 to plants, and 101−200 to bacteria^[8]^. The amino acid sequence identity of UGTs within a family are usually 40% or higher, and within a subfamily is typically 60% or higher^[5,8]^. Additionally, the establishment of the UGT Nomenclature Committee (https://labs.wsu.edu/ugt) is a significant development.

**Figure 2:**
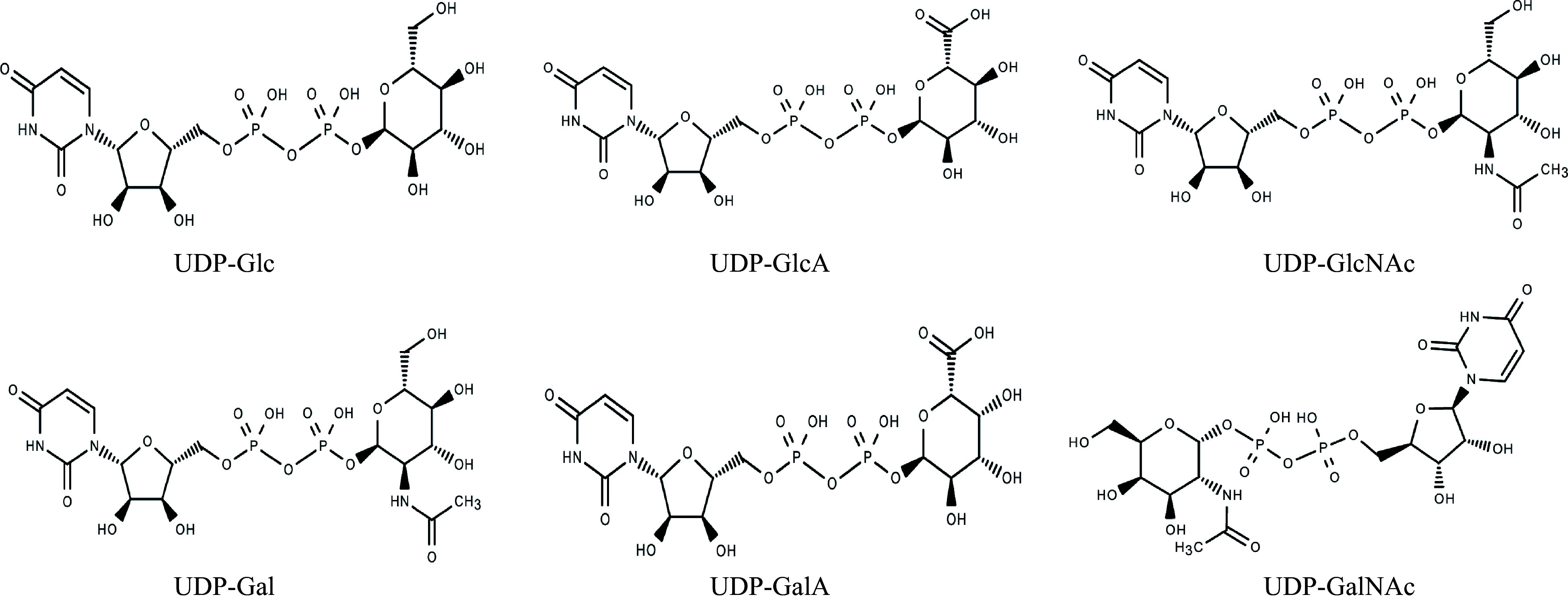
Sugar donors for glycosyltransferase.

Family-1 GTs also referred to as UDP glycosyltransferases (GTs), are the predominant GTs found in plants and play a crucial role in influencing plant development and growth^[8]^. Within these UGT proteins, there exists a conserved consensus sequence near the C-terminal, which spans 44 amino acids and is commonly known as the plant secondary product glycosyltransferase (PSPG) box or signature motif^[6]^. This family displays an inverting mechanism of catalysis and is characterized by a GT-B structural fold. UDP-glucose is the main sugar donor in family 1, followed by UDP-galactose, UDP-rhamnose, UDP-xylose, and UDP-glucuronic acid (Fig. 2)^[2,9]^. UGTs are enzymes that transfer uridine-diphosphate-activated monosaccharides to various compounds. These include anthocyanins, cell wall components, fatty acids, flavonoids, glucosinolates, and phenylpropanoids^[10−14]^.

A significant number of UGTs have been identified across various plant species. Specifically, 107, 168, and 220 UGTs were discovered within the complete genomes of *Arabidopsis thaliana*, peach, and soybean, respectively^[1,15,16]^. Notably, *Quercus suber* possesses 312 UGT family members, representing the largest known UGT family to date. In recent times, extensive research efforts have concentrated on the identification of UGTs in diverse plants. The findings indicated significant variations in the quantities of UGTs among various plant species^[1,15−17]^. Moreover, UGTs play a crucial role in diverse biological processes, regulating the levels of multiple hormones, detoxifying xenobiotics, and stabilizing secondary metabolites chemically^[18−20]^.

It is crucial to comprehend the glycosyltransferase reaction mechanism and glycosides' physiological functions. This understanding is essential for the synthesis of valuable glycosides *in vitro* and the genetic modification of vital crop traits^[2,21]^. The proliferation of UGTs in various species and their independent evolution of functionality sometimes result in inaccurate connections between the structure and function of UGTs. This discrepancy could account for the sluggish advancements in the functional elucidation of UGTs. Despite extensive research on the UGT family over numerous years, only a limited number of UGTs have been thoroughly examined in plant species thus far^[20,22]^. The UGT71 family, for example, belongs to group E, one of the largest UGT groups in plants. UGT71 family members in *Arabidopsis thaliana* have demonstrated the ability to detect various compounds, such as triterpenoids^[1]^, flavonoids^[19]^, and benzoates^[23]^, as well as plant hormones^[24]^.

Predicting substrate specificity is a complicated task because divergent families may recognize the same substrates, and even closely related UGTs can exhibit different affinities for substrates. More and more plant UGTs have been the subject of X-ray crystallography^[25]^, with three-dimensional (3D) structures reported in www.rcsb.org. Protein structural information is essential for the study and understanding of the evolution and catalytic mechanism of glycosyltransferase proteins. The 3D structure of *Arabidopsis thaliana* UGT71C3, as modelled, is depicted in Supplementary Fig. S1. These studies have demonstrated that the secondary and tertiary structures of these proteins are conserved^[26,27]^. An examination of plant UGTs' structure reveals that these enzymes feature two Rossmann folds. The highly conserved C-terminal motif plays a crucial role in binding with activated sugar donors, whereas the variable N-terminal region suggests involvement in sugar receptor binding. Research on the UGT71 family's structure using molecular modeling unveils a region-specific conserved motif at the N-terminus designed for sugar acceptor binding^[28]^. This crucial motif likely hosts several essential residues that might have undergone evolutionary pressure to ensure precise substrate binding.

## Number of UGT genes in plant genomes

Following the rapid progress in plant molecular biology, the molecular-based strategies are being used to isolate genes of interest. The UGT family has been reported to have expanded in land plants, particularly in tracheophytes^[1]^. Numerous genes encoding UGTs with known or potential functions have been discovered in various plant species. Furthermore, recent genome sequencing initiatives have unveiled a multitude of additional UGT genes^[2,10,20]^.

**Figure 3:**
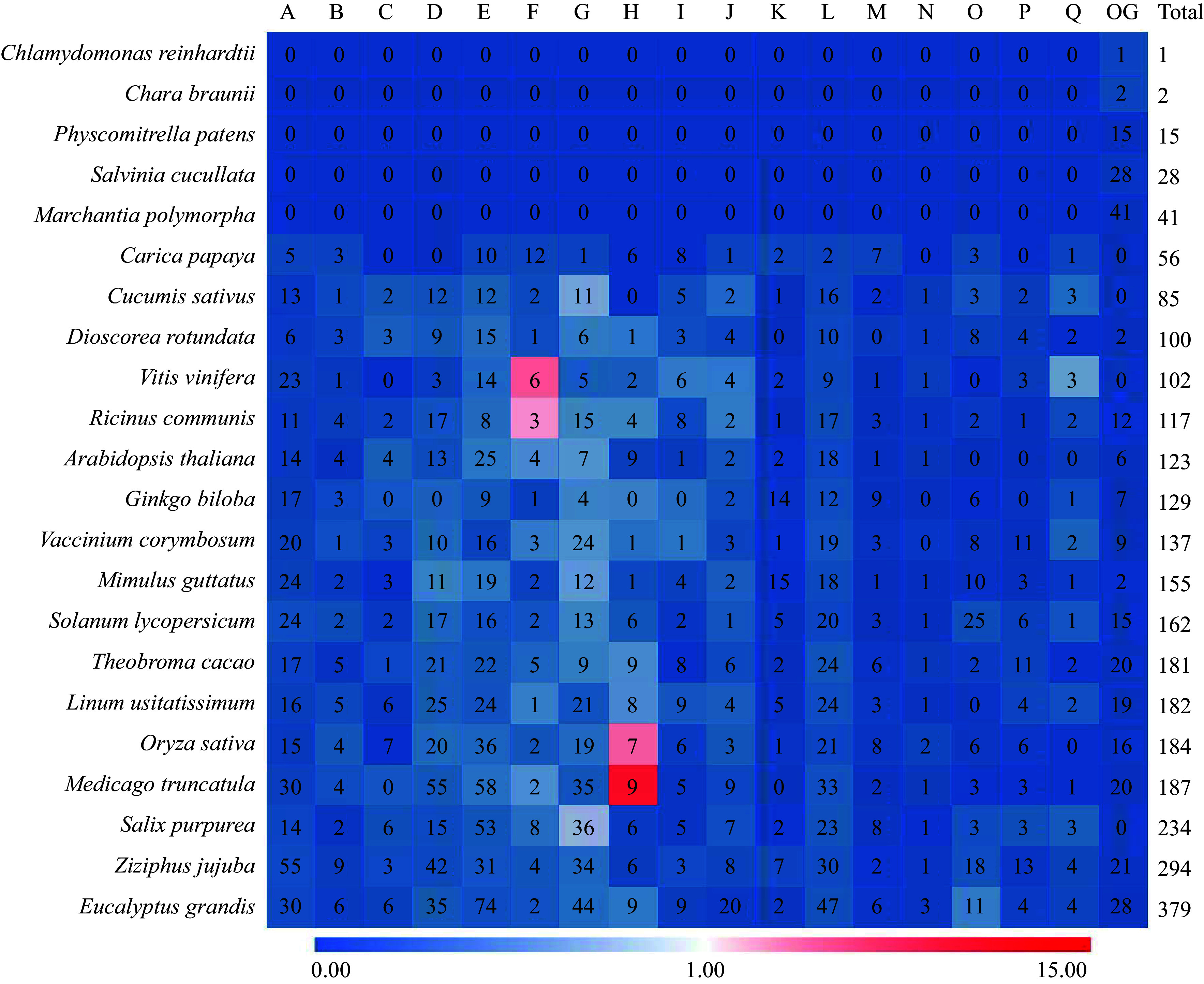
The numbers of total UGTs found in different plants species that have been published in public databases. For each group (i.e. column), the color scale is drawn according to the following ratio (shown in Supplementary Table S1 & S2), the percentage of UGTs group compared with the total UGTs within a given taxon/the percentage of UGTs group compared with the total UGTs within the taxon where the group is first identified.

It was found that the number of UGTs increased gradually during evolution. As shown in Fig. 3 (shown in Supplementary Tables S1 & S2), findings increased from 1, 2, and 15 putative UGTs in the three ancient organisms. These include the following components the single-celled green alga *Chlamydomonas reinhardtii,* the moss *Physcomltrium patens* and the *Chara braunii*, to more than 100 in the arborvitae such as *Selaginella moellendorffii* (gymnosperm), and *Populus trichocarpa* (angiosperm)^[29−32]^. Hypothesizing a swift expansion of the UGT family post differentiation from *P. patens*, it is suggested that this rapid growth persists at varying rates in vascular plants. Notably, the UGT count in vascular plants is 2.7 to 11.5 times greater than in *P. patens*. This observation indicates that the UGT family likely experienced rapid expansion following its divergence from *P. patens* and continues to grow at different velocities in vascular plants^[10]^. Moreover, the phylogenetic group of UGTs seems to remain constant in higher plants. This divergence suggests that the UGT family has been expanding early on in plants, which may demonstrate expansion and contraction of plant UGT groups may have implications for the metabolic activities of specific lines^[33]^. During the evolutionary process of embryophytes, particularly in gymnosperms and shrubby plants, numerous genome-wide, and tandem duplication events have taken place. These events have diversified UGTs, resulting in both sub-functionalization and neo-functionalization^[10]^.

**Figure 4:**
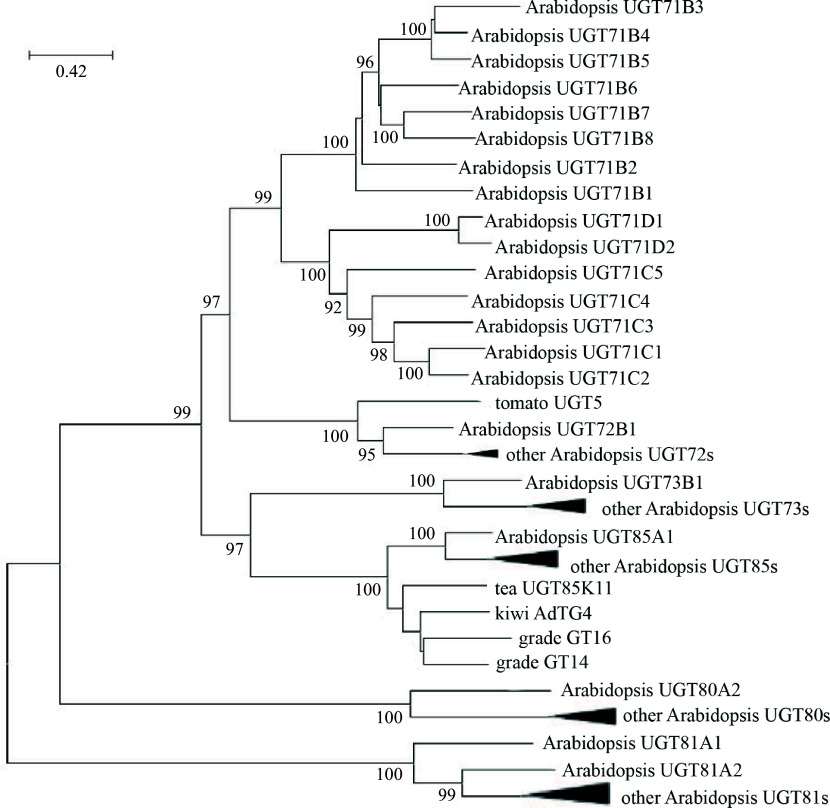
The constructed phylogenetic tree of UGT71s includes glycosyltransferases participating in metabolites such as glycosides. Sequences from different plant species were gathered from the GenBank protein database. UGT genes from *Arabidopsis* were utilized as a reference for UGT families. A tree was built utilizing the maximum-likelihood method with 1,000 bootstrap replications.

The UGT of *Arabidopsis thaliana* was initially utilized to examine evolution, resulting in the categorization of its UGT evolutionary tree into 14 distinct groups, labeled A through N^[5,34]^. Subsequent research has identified two additional groups, O and P, in plants like rice and cucumber^[35]^. Across different species, the number of UGTs in these 14 groups varies significantly. In the course of evolution among higher plants, groups A, D, E, G, and L have experienced rapid expansion, with group E showing the most rapid expansion. Genes within group E make up a notable portion of the UGT superfamily in various species, ranging from 20% to 25%^[1]^. Over time, group E has emerged as the largest subgroup within the GT classification, encompassing UGT71s, UGT72s, and UGT88s. Genes within a group may have evolved convergently in function, while genes with similar functions may have evolved convergently among different groups. To further investigate the evolution of UGT71s, a phylogenetic analysis of *Arabidopsis thaliana* UGTs was conducted to further explore the evolution of UGT71s (Fig. 4). Figure 4 illustrates the phylogenetic tree of UGT enzymes responsible for the biochemical identification of glycosyltransferases, specifically tailored for the metabolites under study. Sequences from various plant species were retrieved from the GenBank protein database. The UGT gene from *Arabidopsis thaliana* served as the reference point for the UGT family. The present research suggests that the glycosylation of flavonoids might be an ancient and conserved function within plant UGTs.

## Substrate recognition specificity of the plant UGT71 family

The biochemical characterization of UGTs' catalytic activity is crucial in the field of glycobiology due to advancements in high-throughput technologies and the identification of new UGTs in plant genomes. With the development of faster and more efficient testing methods, researchers can study the functions and properties of UGT enzymes more comprehensively^[36]^. The discovery of novel UGTs in plant genomes has broadened the scope of glycobiology, shedding light on the diverse roles these enzymes play in biological processes. The study of UGTs' catalytic activity is essential for understanding their role in various biochemical pathways and metabolic processes. By characterizing the enzymatic functions of UGTs, researchers can elucidate their substrate specificity, reaction mechanisms, and potential physiological significance. This information is valuable for developing strategies to modulate UGT activity for applications in agriculture, medicine, and biotechnology. In conclusion, the biochemical characterization of UGTs has contributed significantly to the field of glycobiology, providing insights into the functions and properties of these enzymes in plant genomes. With the use of high-throughput technologies and the discovery of new UGTs, researchers have been able to expand their knowledge of UGT catalytic activity and its implications for biological systems. This research lays the foundation for further exploration of UGT enzymes and their potential applications in various fields.

Advances in bioinformatics have also made significant contributions to the identification of plant UGTs. Numerous conserved motifs have been identified using different motif discovery tools, revealing the PSPG box as a common consensus sequence among UGTs. Additionally, *in silico* strategies have been employed to utilize the increasing amount of analytical and biochemical data on UGTs to predict substrate specificity and biological function^[36−38]^. The 3D structure of an enzyme can be used to predict substrate specificity^[39]^. Future advancements in prediction tools are expected through the utilization of identified 3D structures of UGTs. Despite the isolation of various UGTs from different sources in recent years using these structures, challenges remain in their production, purification, and crystallization^[39]^. Studies have shown that UGT71s are tolerant to substrates *in vitro*, especially to acceptor substrates, whereas they have high specificity for donor substrates^[40]^. UGT71 members utilize triterpenoids, flavonoids, and ABA for substrates. Table 1 summarizes the different known *in vitro* substrates for recombinant UGT71s protein.

**Table 1:** The impact on the expression/activity of UGT71s in plants with known substrate(s).

UGT isoform	Plant species	Substrate(s)	Action	Physiological effects	Ref.
UGT71C5	*Arabidopsis thaliana*	ABA	Knockdown	Drought tolerance and delayed seed germination	[41]
UGT71B6	*Arabidopsis thaliana*	ABA	Overexpression	Higher tolerance to salt, freezing and drought stresses	[42]
UGT71B7	*Arabidopsis thaliana*	ABA	Knockdown	Drought tolerance during germination	[42]
UGT71B8	*Arabidopsis thaliana*	ABA	Knockdown	Drought tolerance during germination	[42]
UGT71C3	*Arabidopsis thaliana*	MeSA	Knockout	*Pseudomonas syringae* infection resistance and increase in MeSA and SA levels	[39]
FaGT6	*Fragaria × ananassa*	Quercetin and kaempferol	*In vitro* catalytic activities	Taking over an additional role in the detoxification of xenobiotics	[43]
UGT71W2	*Fragaria × ananassa*	1-naphthol	Silenced	Increasing stability and water solubility of natural products	[40]
UGT71K3	*Fragaria × ananassa*	Acylphloroglucinol	Silenced	Improving fruit quality	[44]
UGT71A33/34	*Fragaria × ananassa*	3-hydroxycoumarin	*In vitro* catalytic activities	Contributing to the glycosylation of flavonols, xenobiotics	[40]
UGT71A59	*Camellia sinensis*	Eugenol	*In vitro* and *in vivo* catalytic activities	Enhancing cold and drought tolerance of tea plants	[45]
UGT71C1	*Arabidopsis thaliana*	Lignan	Knockout	Increased resistance to oxidative stress	[46]
UGT71C4	*Cotton*	Naringenin	Overexpression	Controls the flux of phenylpropanoid metabolism	[47]
UGT71B1	*Arabidopsis thaliana*	Flavonoids	*In vitro* catalytic activities	Enhancement of metabolite content Increase in their stable storage in plants	[46]
MtUGT71G1	*Medicago truncatula*	Hederagenin	Mutation	Beneficial to plant defence	[48]
UGT71K1	*Malus × domestica*	Phloretin	*In vitro* catalytic activities	Enhance plant disease resistance	[49]
PgUGT1/2	*Panax ginseng*	Ginsenosides	*In vitro* catalytic activities	Enhancement of metabolite content	[48]
UGT71A27	*Panax ginseng*	Dammarane	*In vitro* catalytic activities	Increased stable storage in plants	[48]
MpMUGT3	*Mentha × piperita*	Menthol	*In vitro* catalytic activities	Protects cells from terpenoid toxicity	[50]
CtUGT3	*Carthamus* *tinctorius L.*	Kaempferol	Overexpression	Enhancing the content of medicinal kaempferol glycosides	[51]

### UGT71s glycosylates triterpenoids biosynthetic from plants

The water solubility and biological activity of triterpenoids are altered by glycosylation. The UGT71 family has a very large active site that interacts efficiently with hydrophobic terpenoid structures and it also catalyzes the glycotransferase of triterpenoids. Concerning hydrophobic terpenoid, the *MtUGT71G1* has glycosylation activity towards triterpenoids. The binding pocket in *MtUGT71G1* is primarily made up of amino acids at the N-terminal, along with some residues toward the end of the C-terminus close to where the sugar donor binds^[52]^. In *MtUGT71G1*, the loop is situated at a different location structurally, creating a larger, open pocket that enhances the ability to bind and recognize bulky triterpenoids. By replacing or mutating residues in the binding pocket and creating UGT chimeras through exchanging structural domains, it is possible to enhance its catalytic properties and adjust its specificity towards different substrates. Altering the enzyme structure can effectively influence UGTs and their glycosylation patterns^[52]^. In Panax ginseng, both *PgUGT1* and *PgUGT2*, which are part of the UGT71 family, exhibit biochemical activity on ginsenosides^[53]^. UGTs involved in the biosynthesis of dammarane saponins have been found in ginseng. Notably, *UGT71A27* catalyzes the addition of a glucose molecule to the hydroxyl group at the C-20 position in dammarane diol-II, resulting in the formation of compound K^[54]^.

### Involved in plant flavonoids

Flavonoids usually exist as α or β glycosides, and glycosylation is one of the main factors that bring about the structural diversification of flavonoids^[44]^. UGT71s can be classified into flavonoid 3-O-glycosyltransferases, 7-O-glycosyltransferases, etc., depending on the modification sites in the modification of flavonoid compounds. Concerning flavonoids, in the UGT71s family, *FaGT6* of strawberry *(Fragaria ananassa)* catalyzes the formation of 3-O-glucosides and small amounts of 7-O-, 3'-O-monoglucosides, and diglucosides from quercetin, a flavonol represented by quercetin, and also accepts a variety of flavonoids, hydroxycoumarins, and naphthol as substrates^[55]^. In strawberry (*F.* × *ananassa*), the *UGT71W2* enzyme exhibited the highest level of activity towards 1-naphthol, whereas *UGT71A33*, *UGT71A34a/b*, and *UGT71A35* enzymes had a preference for 3-hydroxycoumarin. Additionally, these enzymes were found to produce 3- and 7-O-glucosides, as well as diglucosides, from flavonols. Furthermore, radiochemical analysis indicated that *UGT71A33*, *UGT71A34*, and *UGT71A35* enzymes acted on the hydroxyl groups at positions 3 and 7 of the flavonols. In contrast, UGT71W2 was unable to catalyze the formation of the glucoside at position 7^[3,34,56]^. The *Arabidopsis thaliana* *UGT71C1* rapidly converts quercetin diglucosides glycosides to 3'-O- and 7-O-monoglucosides. These compounds then act as substrates to produce 7, 3'-di-O-glucosides. The *ugt71c1* mutants show significantly decreased levels of quercetin 3,7-O glucoside and kaempferol 3,7-O glucoside in comparison to the wild type, with reductions of 25% and 70% respectively^[57]^. Additionally, there is a notable decrease in the content of lariciresinol and pinoresinol-glucosides in these mutants^[58]^. In addition to quercetin and lignoceroside, recombinant *UGT71C1* also glycosylates lignans and turpentines^[48]^. Isomeric larch alcohol is an isomer of larch alcohol and its 4'-β-D-glucosideform has lower antioxidant properties than its glycosidic form^[59]^. In safflower (*Carthamus tinctorius* L.), *CtUGT3* may be involved in regulating the biosynthesis of flavonol-3-O-glucoside in both lines^[60]^.

### Phytohormones

The glycosylation modification of phytohormones refers to the process by which a hormone forms a complex with a sugar molecule, thereby regulating plant growth and development through synergistic interactions with the glycosylated product^[35]^. Phytohormones are known to engage in several different types of conjugation reactions, including glycosylation. This process has been observed in substances derived from various phytohormones such as abscisic acid (ABA), gibberellins, strigolactones, cytokinins, auxins, brassinosteroids, salicylic acid, and jasmonic acid. This process contributes to the functional diversity and regulatory capacity of phytohormones in plants, playing a vital role in plant physiology and development. Glycosylation of phytohormones is a complex and dynamic process that helps to modulate the effects of these signaling molecules on plant growth and responses to environmental stimuli^[61−63]^. Among the hormones, plant UGT71s are primarily responsible for glycosylation of ABA. The role of UGT71s in abscisic acid (ABA) metabolism has been thoroughly investigated in the model organism *Arabidopsis thaliana*. Specifically, *UGT71B6*, *UGT71B7*, and *UGT71B8* enzymes catalyze the production of ABA-glucosyl ester (ABA-GE). The genes encoding these enzymes are upregulated by ABA, as well as by abiotic stresses such as high salinity and osmotic stress^[19,24]^. Furthermore, experimental interference with the expression of *UGT71C5* in *Arabidopsis thaliana* has been shown to increase the concentration of free ABA. This increase in free ABA leads to enhanced drought tolerance and delays in seed germination. Biochemical analyses have demonstrated that *UGT71C5* can glucosylate ABA both *in vitro* and *in vivo*, highlighting its involvement in the plant's resistance to drought stress^[41]^.

On the other hand, UGT71s indirectly affect SA levels. In addition to directly controlling SA levels in plants, UGT can glycosylate other compounds that in turn affect SA levels^[64]^. These compounds encompass salicylic acid (SA) metabolites like *UGT71C3*, which methylates and glycosylates salicylate (MeSA) in *Arabidopsis thaliana*. This process is predominantly triggered in leaf tissues by pathogens, such as *Pseudomonas syringae pv.* tomato strain DC3000 with the avrRpt2 gene (Pst DC3000/avrRpt2)^[65]^.

## Functional features of the UGT71 family in plant secondary metabolites

**Figure 5:**
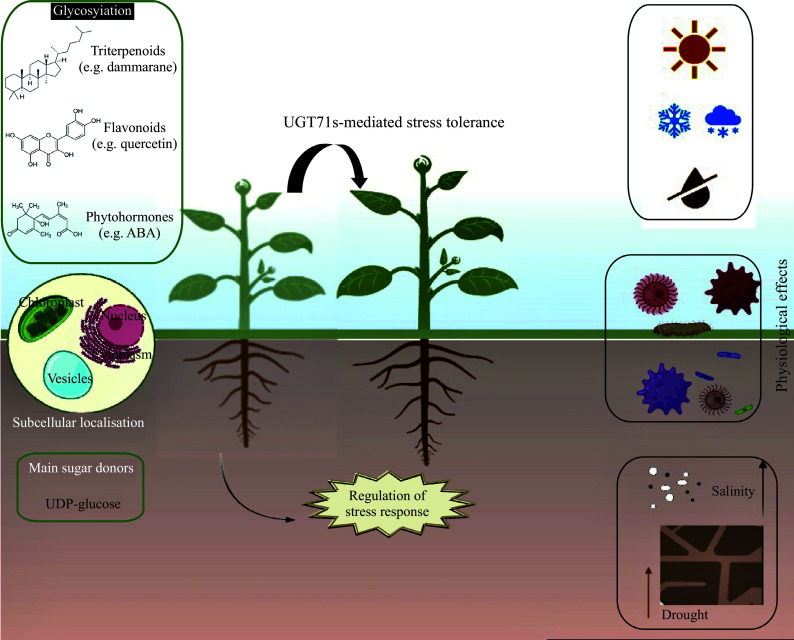
Roles of UGT71s in plant response to stresses. Among the biological processes in which UGT71s are involved are glycosylation of secondary metabolites (mainly triterpenoids and flavonoids) and glycosylation of phytohormones. In addition, this chemical modification plays a role in the response to plant fitness, thus helping plants to adapt to changing environments.

Plant secondary metabolites are diversified through the cooperation of UGTs with acyltransferases, methyltransferases, and cytochromes P450s, among others, thereby altering their physicochemical properties^[20,36,65]^. The activity of UGT could be significant for the maintenance of homeostasis in different metabolic pathways. The essential regulation helps in maintaining redox stability, particularly when dealing with the equilibrium between the aglycone and glycosylated forms of molecules^[38]^. The wide variety of enzyme functions in secondary metabolites offers plants the adaptability needed for survival in a dynamic environment^[35]^. From an evolutionary perspective, the substrates for each glycosyltransferase have become increasingly specialized, and each enzyme has retained its irreplaceability through natural selection, resulting in an evolutionary diversity of secondary metabolites^[65]^. The UGT71s that glycosylate plants triterpenoids, flavonoids, hormones and as such they may have a critical role in regulating these processes (Table 1). Playing a role in improving the water solubility, inactivation or detoxification of natural products^[10]^, and may contribute to redox homeostasis through various biochemical mechanisms. In this section, the process of glycosylation in plant secondary metabolites is analyzed, specifically focusing on the role of UGT71 enzymes. These enzymes play a crucial role in modifying secondary metabolites through the addition of sugar molecules, which can impact a plant's response to environmental stressors. Glycosylation catalyzed by UGT71s has been shown to regulate the accumulation and distribution of secondary metabolites within plants, leading to changes in their defense mechanisms against external threats (Fig. 5). Furthermore, studies have demonstrated that glycosylation mediated by UGT71s enzymes can enhance the stability and solubility of secondary metabolites, making them more available for defense responses. This process also influences the production of volatile compounds that play a role in plant interactions with other organisms in their environment. By understanding the mechanisms of glycosylation involving UGT71s, researchers can gain insights into how plants adapt and respond to different stress conditions, ultimately contributing to the development of strategies for improving crop resilience and productivity.

### Possible roles of UGT71s in triterpenoids biosynthetic

Triterpenoids are a class of secondary metabolites associated with general defense and stress responses^[66]^. Triterpenoids are a significant class of plant secondary metabolites featuring a 30-carbon atom basic nucleus. They are widely distributed in nature as either free or sugar-bound glycosides or esters. Glycosylation plays a significant role in modifying the physicochemical properties and biological activities of triterpenes in plants, enhancing water solubility and influencing the biological activity of triterpenoids^[67]^. Additionally, it has been identified as a key element in plant defense mechanisms^[50]^. Glycosylation of hydroxyl and/or carboxyl groups in triterpenoids results in the formation of various triterpenoid derivatives. Despite the identification of a few UGT enzymes capable of glycosylating triterpene aglycones, many UGTs have not been thoroughly studied in terms of their biochemical functions and substrate specificities. UGT71s exhibit significant structural diversity in these important molecules by attaching monosaccharides to triterpene aglycones and triterpenoid glycosides, enabling the modification of their water solubility and biological properties.

Constitutive terpenoid saponins were found to possess insecticidal and antifungal activities, and glycosylation of triterpenoids was found to be a key component of plant defense mechanisms^[50]^. Recently, *MpMUGT3a* and *MpMUGT3b* from *peppermint* × *pepper* and *lycopene* have recently been reported to have detoxifying effects on the menthol and geranic acid, suggesting that glycosyltransferases can protect cells from terpenoid toxicity^[68]^. And the antifungal effects of triterpenoid saponins are during plant growth associated with the glycoside molecule, where esterification of the hydroxyl group results in altered activity^[69]^. During plant growth, the accumulation of triterpenoids biosynthetic varies depending on the environment, which depends on the conservation needs of plant organs and tissues. For instance, the removal or modification of sugar residues can lead to a decrease in biological activity, while resistance to fungal pathogens can be attributed to glycosylation at specific carbon sites. Hydrolytic enzymes known as avenacinase, produced by root-infected rhizobacteria (*Gaeumannomyces graminis*), exhibit β-glucosidase activity targeting the C-3 chain to detoxify vinculin saponins^[41]^. Specific saponins tend to accumulate during root and fruit development in saponin-producing plants like ginseng and red ginseng^[70,71]^. This phenomenon has been associated with the catalytic function of specific UGTs during these biological processes, with saponin trans-activation observed in *Dioscorea pseudojaponica* (yam) tubers and oat root epidermis when exposed to soil-borne fungi^[48]^. Therefore, it can be inferred that the biosynthetic activity of UGT71s plays a crucial role in physiological processes and defense responses, resulting in the accumulation of specific terpenoid biosynthesis.

### Regulation of flavonoids

The wide substrate specificity demonstrated by most UGT71s *in vitro* complicates the identification of their authentic substrates *in vivo*^[72]^. Physiological glycoside libraries containing a variety of natural glycosides offer a valuable approach to uncovering potential natural substrates of UGTs^[73]^. Accumulation of flavonoids significantly increases the tolerance of various plants to oxidative stress caused by intense irradiation or drought^[74]^, and producing a large number of glycosides found in plants.

Plant flavonoid glycosides serve as the primary active components in several key traditional Chinese medicines. Glycosylation typically occurs as the final stage in the synthesis of flavonoid glycosides. This process not only alters the polarity of flavonoid compounds but also impacts the pharmacodynamic activity and pharmacokinetics of flavonoids. Flavonoid glycosides are considered to be more potent antioxidants compared to flavonoids^[75]^. Studies have shown that *UGT71C1* can glycosylate the 3-OH of flavonoids *in vitro*. The *ugt71c1* mutant shows decreased levels of quercetin 3,7-O-glucoside compared to the wild-type. Quercetin in the vacuole, chloroplast, and nuclear compartments act to scavenge reactive oxygen species (ROS) through various mechanisms^[76,77]^. Specifically, in the vacuole, quercetin reduces H_2_O_2_ to H_2_O in a peroxidase-dependent manner^[78]^. These radicals are then recycled to their reduced form via ascorbate, enabling the reduction of further H_2_O_2_ molecules^[79]^. Flavonoids exhibit efficient singlet oxygen scavenging activity when located in chloroplasts, and protect DNA against oxidative damage when present in the nucleus^[80,81]^. The primary structural characteristic responsible for the free radical scavenging capacity of flavonoids is the highly reactive nature of their hydroxyl substituents^[82]^.

$ \rm {Flavonoids} \;{\text ——}\; {OH+R{\text'} \to Flavonoids} \;{\text ——}\; {O{\text'}+RH} $

The results indicate that UGT71C1 is involved in glycosylation pathways related to flavonoids under oxidative stress conditions, and modifies the plant cellular redox scavenging potential by showing increased resistance to oxidative stress. This demonstrates the role of UGT71s in redox homeostasis.

Strawberry glycoside extracts were used as physiological libraries by enzymatic hydrolysis to search for *in vivo* substrates of LC-MS analyses determined that 3-hydroxycoumarin was the natural substrate for *UGT71A33*, *UGT71A34a,* and *UGT71A35*, and quercetin was a natural substrate for *UGT71A34a*^[40]^. It was found that the function of *UGT71W2* in plants was analyzed by agroinfiltration in fruiting discs of *F.* × *ananassa* *cv*. It was down-regulated *UGT71W2* transcripts using RNAi as a medium. This experiment successfully down-regulated the level of *UGT71W2* transcripts in strawberry fruiting discs by injecting *Agrobacterium tumefaciens* carrying the *p9U10*-*UGT71W2* vector. UGT71W2 was found to have no impact on the color of strawberry fruits, however, it did lead to a significant decrease in the levels of kaempferol glucoside and kaempferol-3-(60-coumaroyl) glucoside, to some extent. In contrast, it is hypothesized that possibly excess 4-coumaroyl molecules are diverted to hydroxycinnamoyl glucoside biosynthesis, with higher levels of 4-coumaroylglucoside accumulation^[40,44]^. Combined with the results of library analyses as well as targeted and untargeted metabolite analyses performed on agroinfiltrated fruits demonstrates that *UGT71W2* may contribute to the glucosylation of 1-naphthol, estrogens in plants. These findings further illuminate the specificity of plant UGT71s substrates, showcasing both their limited and extensive plasticity to metabolites of similar structures. This phenomenon ultimately enables the relatively small yet diverse group of UGTs to effectively produce the wide array of glycosides present in plants.

### UGT71s are involved in the glycosylation of phytohormones in response to stress

#### Glycosylation of phytohormones in response to abiotic stress

Phytohormones play a crucial role in mediating plant stress responses by regulating the balance between inactive glycosides and their active forms, enabling plants to efficiently adapt to environmental fluctuations^[83,84]^. The main phytohormone for glycosylation regulation mediated by UGT71s is abscisic acid (ABA)^[20]^. UGT71s play a role in glycosylating phytohormones in response to abiotic stress. As sessile organisms, plants must adapt to changing environments, which may include exposure to common abiotic stresses such as drought, soil salinity, and extreme temperatures^[85]^. Maintaining homeostasis and glycosylation of ABA is crucial for enhancing tolerance to water or salt stress and reducing oxidative damage.

ABA performs a critical role in dealing with abiotic stresses^[86]^. UGTs control the quantity of unconjugated ABA in two ways: First, they create an inactive storage form called ABA glucosyl ester (ABE-GE), which swiftly breaks down to produce active, unconjugated ABA^[87]^. ABA-GE is sequestered in plant vesicles and the apoplastic space^[88]^. The pathway for ABA synthesis involves the straightforward one-step hydrolysis of glucose-conjugated ABA (ABA-GE) to ABA by β-glucosidases, specifically AtBG1 which are localized in the endoplasmic reticulum and vacuole. The *AtBG1* is localized to the ER, suggesting that ABA-GE may require input to the ER. Dehydration stress can trigger the transportation of ABA-GE across the ER membrane to the ER^[89]^. This process is likely to be highly regulated to meet the plant's demand for ABA, as AtBG1 and its substrate ABA-GE are usually stored in different compartments within the cell. They are only brought together when elevated levels of ABA are needed in response to abiotic stress^[87]^.

Several experiments have demonstrated that altering the expression of specific UGTs, responsible for ABA glycosylation, notably affects plant fitness under unfavorable abiotic conditions. Disrupting the expression of *UGT71C5* in *Arabidopsis thaliana* led to an increase in drought tolerance, delayed seed germination, and raised free ABA concentration. On the other hand, overexpression negatively affected all three factors^[41]^. Manipulating the expression of ABA-glycosylating UGTs can lead to varying phenotypic outcomes based on the plant's maturity. Silencing *UGT71B6*, *UGT71B7*, and *UGT71B8* in *Arabidopsis thaliana* resulted in growth defects and decreased salt tolerance in adult plants. Conversely, it improved tolerance to osmotic stress induced by drought, salinity, or cold stress^[90]^, as well as enhanced drought tolerance during germination^[42]^. Furthermore, these proteins exhibited the ability to glucosylate different structural analogs of ABA to different extents^[91]^. The products were identified as glucoseesters^[91]^. Overexpression of *UGT71B6* during osmotic stress conditions suppressed the expression of genes related to drought tolerance and osmotic stress. *UGT71B6*, along with its homologs *UGT71B7* and *UGT71B8*, modulate ABA levels in living organisms and are crucial for plant cellular responses to dehydration and osmotic stress, as well as for plant germination and growth. Thus, it may be assumed that the UGT71s have a biological function of regulating ABA levels to maintain optimal growth conditions.

#### Glycosylation of phytohormones in response to biotic stress

In addition to coping with abiotic stresses, plants are also capable of resisting and adapting to biotic stresses^[92]^. Pests and pathogens frequently attack plants by either feeding on plant parts or injecting toxins into the plant^[93]^. Toxins released by pathogens like bacteria and fungi can cause or worsen plant diseases^[94]^. Plant UGTs catalyze the glycosylation of toxins, providing protection to the plant and potentially enhancing the affinity of these compounds for membrane-bound transporters, facilitating toxin export^[95]^. Additionally, UGT71s can indirectly regulate biotic stress responses by glycosylating plant hormones, playing a significant role in plant defense mechanisms.

In *Arabidopsis thaliana*, *UGT71C3* was identified as an enzyme that transfers glucose to MeSA, making it a MeSA glucosyltransferase. According to biochemical analyses, *UGT71C3* has a high enzymatic activity towards MeSA, and can produce MeSA glucosides both *in vitro* and *in vivo*. After Pst DC3000/avr Rpt2 primary pathogen infection, the *ugt71c3* knockout mutant displayed greater systemic resistance to secondary infection by pathogens, while the *UGT71C3* overexpressing lines showed compromised systemic resistance compared to wild-type plants^[96]^. Similarly, localized primary infection of leaves led to significantly higher levels of systemic MeSA and SA accumulation in the *ugt71c3* knockout mutant than in wild-type plants, while the *UGT71C3* overexpressing strain accumulated lower levels of MeSA and SA. Furthermore, the *UGT71C3* overexpressing lines presented much higher levels of Me SAG than wild-type plants^[97]^. Induction of pathogens and MeSA up-regulates the expression of *UGT71C3*, leading to accelerated glucosylation of MeSA, which results in less MeSA being translocated to uninoculated systemic tissues. In systemic tissues, *UGT71C3* further glucosylates MeSA, resulting in reduced MeSA levels and consequently, reduced levels of SA translocated from MeSA. This ultimately leads to a reduction in the expression level of pathogen-associated proteins, weakening the defense response^[56,98]^.

## Conclusions and perspectives

Glycosylation, catalyzed by UGTs, is a modification reaction that is necessary for plant cell growth, development, and metabolic homeostasis^[47]^. It plays an important role in defense, hormone regulation, xenobiotic modification and detoxification of pollutants, biosynthesis of secondary metabolites and plant-microbe interactions. Therefore, UGTs are closely related to life processes such as plant seed germination, growth, flowering, and fruiting^[6]^. In recent years, the completion and improvement of genome sequencing and the assembly of plant species, the revelation of molecular functions by forward and reverse genetics, and the determination of enzyme substrates and products by biochemical reactions have led to the discovery, identification, and clarification of an increasing number of UGT genes. This has established a robust theoretical foundation for the further elucidation of the biological functions of the UGT family^[11]^. Nevertheless, as a superfamily, the specific working model and mechanism of action of the majority of UGT family members remain unclear. This paper presents a summary of the main properties of the UGT71family in plants, shown in Fig. 5. The substrates of UGT71s enzymes are mainly UDP-glucose as a sugar donor, and they play an important role by glycosylating secondary metabolites and glycosylating phytohormones (Fig. 5). Firstly, with regard to secondary metabolites, in particular, some members of the UGT71 family have been demonstrated to modify triterpenoids and flavonoids, thereby facilitating the biosynthesis of secondary metabolites and improving plant stress tolerance, among other effects. With respect to hormones, UGT71s mainly modify ABA and SA, which play regulatory roles in biotic and abiotic stresses.

UGTs have the potential to play a pivotal role in a range of bioremediation strategies and agriculture on marginal lands^[16]^. However, further studies are required to elucidate the intricate interplay of stress pathways in plants and the underlying molecular mechanisms. This will facilitate the development of safe and efficient biotechnological solutions^[6,16]^. The identification of additional UGT71s in plants will further promote research on the structure and function of UGTs, which have important functional roles in hormonal pathways and in enhancing stress tolerance in plants. Furthermore, they have been found to have the potential to safeguard and improve crop yields. Their involvement in regulating secondary metabolites in plants has also given them the potential to enhance the quality of crops. On the other hand, it has been reported that the majority of chemical synthesis of flavonoids necessitates the use of toxic chemicals and extreme reaction conditions. However, the utilization of plant UGT71s to catalyze *in vitro* the conversion of UGT71s into flavonoid glycoside derivatives through glycosylation represents a promising avenue for the synthesis of flavonoids. This is expected to resolve the issue of synthesizing active flavonoids artificially and to pave the way for the utilization of analogous derivatives as synthetic drug ingredients. UGT71s regulate the accumulation of plant metabolites by participating in the glycosylation pathway of various compounds, thus protecting plants from a multitude of biotic and abiotic stresses, which is of great significance for crop genetic improvement and provides a novel direction for the cultivation of new varieties with high resistance to diseases.

The functional study of UGT71 family genes provides a robust theoretical foundation for future crop improvement. Firstly, UGT71 genes that confer resistance to abiotic stress can be cloned and subsequently overexpressed in plants, resulting in crops that exhibit enhanced resilience to such stresses and improved yields. This approach lays the groundwork for genetic breeding and CRISPR/Cas9 applications in agricultural and forestry crops, while also offering essential genetic resources for developing plants with increased resistance to adversity. Secondly, genetic engineering of the UGT71 family can enhance the yield of metabolites with nutritional and health benefits, such as flavonoids, thereby improving the nutritional and health qualities of food products to better meet consumer demands. Additionally, the UGT71 gene family can be utilized to reduce pesticide residues and contaminants in crops, thereby improving food safety. This represents a critical reference point for the implementation of design breeding strategies in the agricultural and forestry sectors. Furthermore, in the context of global warming and the associated deterioration of growing conditions due to climate change, it has the potential to enhance the adaptability and resilience of plants.

Nevertheless, a significant amount of work remains to be done, including *in vivo* studies involving metabolite profiling of plants overexpressing UGTs and knockout plants lacking individual UGTs. These studies should also examine tissue and subcellular specificity, along with regulatory promoter elements for metabolite accumulation. To gain a comprehensive understanding of the biological roles of recombinant UGTs, it will be necessary to combine these studies with *in vitro* catalytic analyses of recombinant UGTs.

## SUPPLEMENTARY DATA

Supplementary data to this article can be found online.

## Data Availability

All data generated or analyzed during this study are included in this published article and its supplementary information files.

## References

[1] (2012). A genome-wide phylogenetic reconstruction of family 1 UDP-glycosyltransferases revealed the expansion of the family during the adaptation of plants to life on land. The Plant Journal.

[2] (2006). Glycosyltransferases of lipophilic small molecules. Annual Review of Plant Biology.

[3] (2001). Glycosyltransferases in secondary plant metabolism: tranquilizers and stimulant controllers. Planta.

[4] (2019). The UDP-glycosyltransferase (UGT) superfamily: new members, new functions, and novel paradigms. Physiological Reviews.

[5] (2001). Higher plant glycosyltransferases. Genome Biology.

[6] (2011). An evolutionary view of functional diversity in family 1 glycosyltransferases. The Plant Journal.

[b7] 7Sun S, Dai T, Wang Z, Chou J, Chao Q, et al. 2021. Projected increases in population exposure of daily climate extremes in Eastern China by 2050. Advances in Climate Change Research

[8] (1997). The UDP glycosyltransferase gene superfamily: recommended nomenclature update based on evolutionary divergence. Pharmacogenetics.

[9] (2019). New steps in mucilage biosynthesis revealed by analysis of the transcriptome of the UDP-rhamnose/UDP-galactose transporter 2 mutant. Journal of Experimental Botany.

[10] (2012). Two glycosyltransferases involved in anthocyanin modification delineated by transcriptome independent component analysis in *Arabidopsis thaliana*. The Plant Journal.

[11] (2016). UDP-glycosyltransferase 72B1 catalyzes the glucose conjugation of monolignols and is essential for the normal cell wall lignification in *Arabidopsis thaliana*. The Plant Journal.

[12] (2016). Structural insights and membrane binding properties of MGD1, the major galactolipid synthase in plants. The Plant Journal.

[13] (2014). Comparative analysis of *Arabidopsis* UGT74 glucosyltransferases reveals a special role of UGT74C1 in glucosinolate biosynthesis. The Plant Journal.

[14] (2007). The hyper-fluorescent trichome phenotype of the *brt1* mutant of *Arabidopsis* is the result of a defect in a sinapic acid: UDPG glucosyltransferase. The Plant Journal.

[15] (2019). UDP-glucosyltransferase PpUGT85A2 controls volatile glycosylation in peach. Journal of Experimental Botany.

[16] (2017). Genome-wide identification and functional characterization of UDP-glucosyltransferase genes involved in flavonoid biosynthesis in *Glycine max*. Plant and Cell Physiology.

[17] (2005). The UGT73C5 of *Arabidopsis thaliana* glucosylates brassinosteroids. Proceedings of the National Academy of Sciences of the United States of America.

[18] (2011). *N*-glucosyltransferase UGT76C2 is involved in cytokinin homeostasis and cytokinin response in *Arabidopsis thaliana*. Plant and Cell Physiology.

[19] (2004). A class of plant glycosyltransferases involved in cellular homeostasis. The EMBO Journal.

[20] (2005). Plant secondary metabolism glycosyltransferases: the emerging functional analysis. Trends in Plant Science.

[21] (2010). Functional differentiation of the glycosyltransferases that contribute to the chemical diversity of bioactive flavonol glycosides in grapevines (*Vitis vinifera*). The Plant Cell.

[22] (2005). Metabolic engineering of dhurrin in transgenic *Arabidopsis* plants with marginal inadvertent effects on the metabolome and transcriptome. Proceedings of the National Academy of Sciences of the United States of America.

[23] (2002). The activity of *Arabidopsis* glycosyltransferases toward salicylic acid, 4-hydroxybenzoic acid, and other benzoates. Journal of Biological Chemistry.

[24] (2020). Metabolic profiling and transcriptome analysis of mulberry leaves provide insights into flavonoid biosynthesis. Journal of Agricultural and Food Chemistry.

[25] (2015). Insights into the UDP-sugar selectivities of human UDP-glycosyltransferases (UGT): a molecular modeling perspective. Drug Metabolism Reviews.

[26] (2017). Identification of a residue responsible for UDP-sugar donor selectivity of a dihydroxybenzoic acid glycosyltransferase from *Arabidopsis* natural accessions. The Plant Journal.

[27] (2002). Remarkable structural similarities between diverse glycosyltransferases. Chemistry & Biology.

[28] (2018). Analysis of two new Arabinosyltransferases belonging to the carbohydrate-active enzyme (CAZY) glycosyl transferase Family1 provides insights into disease resistance and sugar donor specificity. The Plant Cell.

[29] (2019). Phylogenomic analysis of UDP-dependent glycosyltransferases provides insights into the evolutionary landscape of glycosylation in plant metabolism. The Plant Journal.

[30] (2016). Functional characterization of UDP-apiose synthases from bryophytes and green algae provides insight into the appearance of apiose-containing glycans during plant evolution. Journal of Biological Chemistry.

[31] (2017). Tomato UDP-glucose sterol glycosyltransferases: a family of developmental and stress regulated genes that encode cytosolic and membrane-associated forms of the enzyme. Frontiers in Plant Science.

[32] (2022). Genome wide analysis of Family-1 UDP glycosyltransferases in *Populus trichocarpa* specifies abiotic stress responsive glycosylation mechanisms. Genes.

[33] (2018). Whole-genome duplication and plant macroevolution. Trends in Plant Science.

[34] (2001). Phylogenetic analysis of the UDP-glycosyltransferase multigene family of *Arabidopsis thaliana*. Journal of Biological Chemistry.

[35] (2009). The genome of the cucumber, *Cucumis sativus* L. Nature Genetics.

[36] (2018). Functional and informatics analysis enables glycosyltransferase activity prediction. Nature Chemical Biology.

[37] (2016). Plant secondary metabolism linked glycosyltransferases: an update on expanding knowledge and scopes. Biotechnology Advances.

[38] (2008). Glycosyltransferases: structures, functions, and mechanisms. Annual Review of Biochemistry.

[39] (2009). Substrate specificity of plant UDP-dependent glycosyltransferases predicted from crystal structures and homology modeling. Phytochemistry.

[40] (2015). Functional characterization and substrate promiscuity of UGT71 glycosyltransferases from strawberry (*Fragaria* × *ananassa*). Plant and Cell Physiology.

[41] (2015). UDP-glucosyltransferase71C5, a major glucosyltransferase, mediates abscisic acid homeostasis in *Arabidopsis*. Plant Physiology.

[42] (2014). A novel bHLH transcription factor *PebHLH35* from *Populus euphratica* confers drought tolerance through regulating stomatal development, photosynthesis and growth in *Arabidopsis*. Biochemical and Biophysical Research Communications.

[43] (2017). Characterization of *UGT716A1* as a multi-substrate UDP: flavonoid glucosyltransferase gene in *Ginkgo biloba*. Frontiers in Plant Science.

[44] (2015). Dietary flavonoid aglycones and their glycosides: which show better biological significance. Critical Reviews in Food Science and Nutrition.

[45] (2022). Eugenol functions as a signal mediating cold and drought tolerance via UGT71A59-mediated glucosylation in tea plants. The Plant Journal.

[46] (2008). Improved resistance to oxidative stress by a loss-of-function mutation in the *Arabidopsis*
*UGT71C1* gene. Molecules and Cells.

[47] (2024). UDP-glucosyltransferase 71C4 controls the flux of phenylpropanoid metabolism to shape cotton seed development. Plant Communications.

[48] (2011). Molecular activities, biosynthesis and evolution of triterpenoid saponins. Phytochemistry.

[49] (2012). Phytozome: a comparative platform for green plant genomics. Nucleic Acids Research.

[b50] 50Mugford ST, Osbourn A. 2012. Saponin synthesis and function. In Isoprenoid Synthesis in Plants and Microorganisms, eds Bach T, Rohmer M. New York: Springer. pp. 405–24.

[51] (2023). Identification and characterization of CtUGT3 as the key player of astragalin biosynthesis in *Carthamus tinctorius* L. Journal of Agricultural and Food Chemistry.

[52] (2009). Structure, mechanism and engineering of plant natural product glycosyltransferases. FEBS Letters.

[53] (2014). Grouping and characterization of putative glycosyltransferase genes from *Panax ginseng* Meyer. Gene.

[54] (2014). Production of bioactive ginsenoside compound K in metabolically engineered yeast. Cell Research.

[55] (2008). Multi-substrate flavonol *O*-glucosyltransferases from strawberry (*Fragaria × ananassa*) achene and receptacle. Journal of Experimental Botany.

[56] (2022). Structure-function relationship of terpenoid glycosyltransferases from plants. Natural Product Reports.

[57] (2020). You want it sweeter: how glycosylation affects plant response to oxidative stress. Frontiers in Plant Science.

[58] (2014). Glucosyltransferase activity of *Arabidopsis* UGT71C1 towards pinoresinol and lariciresinol. Plant Biotechnology.

[59] (2001). Isolation and characterization of novel benzoates, cinnamates, flavonoids, and lignans from Riesling wine and screening for antioxidant activity. Journal of Agricultural and Food Chemistry.

[60] (2016). Expression patterns of three *UGT* genes in different chemotype safflower lines and under MeJA stimulus revealed their potential role in flavonoid biosynthesis. PLoS One.

[61] (2011). Conjugates of abscisic acid, brassinosteroids, ethylene, gibberellins, and jasmonates. Phytochemistry.

[62] (2013). Increased biomass, seed yield and stress tolerance is conferred in *Arabidopsis* by a novel enzyme from the resurrection grass *Sporobolus stapfianus* that glycosylates the strigolactone analogue GR24. PLoS One.

[63] (2019). Intra and extracellular journey of the phytohormone salicylic acid. Frontiers in Plant Science.

[64] (2009). Use of a synthetic salicylic acid analog to investigate the roles of methyl salicylate and its esterases in plant disease resistance. Journal of Biological Chemistry.

[65] (2019). Methyl salicylate glucosylation regulates plant defense signaling and systemic acquired resistance. Plant Physiology.

[66] (2007). The function of terpene natural products in the natural world. Nature Chemical Biology.

[67] (2014). A UDP-glucose: monoterpenol glucosyltransferase adds to the chemical diversity of the grapevine metabolome. Plant Physiology.

[68] (2022). Detoxification of monoterpenes by a family of plant glycosyltransferases. Phytochemistry.

[69] (2010). Ligninolytic and antioxidative enzymes of a marine cyanobacterium *Oscillatoria willei* BDU 130511 during Poly R-478 decolourization. Bioresource Technology.

[70] (2015). Biosynthesis and biotechnological production of ginsenosides. Biotechnology Advances.

[71] (2018). Pharmacological activities of mogrosides. Future Medicinal Chemistry.

[72] (2005). Genomics-based selection and functional characterization of triterpene glycosyltransferases from the model legume *Medicago truncatula*. The Plant Journal.

[73] (2014). Activity-based profiling of a physiologic aglycone library reveals sugar acceptor promiscuity of Family 1 UDP-glucosyltransferases from grape. Plant Physiology.

[74] (2019). Manipulation of oxidative stress responses as a strategy to generate stress-tolerant crops. From damage to signaling to tolerance. Critical Reviews in Biotechnology.

[75] (2005). Structure–antioxidant activity relationships of flavonoids isolated from different plant species. Food Chemistry.

[76] (2012). Flavonoids as antioxidants in plants: location and functional significance. Plant Science.

[77] (2019). RBOH-dependent ROS synthesis and ROS scavenging by plant specialized metabolites to modulate plant development and stress responses. Chemical Research in Toxicology.

[78] (2007). Flavonoid oxidation in plants: from biochemical properties to physiological functions. Trends in Plant Science.

[79] (1997). Flavonoid-peroxidase reaction as a detoxification mechanism of plant cells against H_2_O_2_. Plant Physiology.

[80] (2008). Subcellular localization of flavonoid natural products: a signaling function. Plant Signaling & Behavior.

[81] (2019). Review: ABA, flavonols, and the evolvability of land plants. Plant Science.

[82] (2002). Flavonoid antioxidants: chemistry, metabolism and structure-activity relationships. The Journal of Nutritional Biochemistry.

[83] (2016). Glucosylation of 4-hydroxy-2, 5-dimethyl-3(2H)-furanone, the key strawberry flavor compound in strawberry fruit. Plant Physiology.

[84] (2021). How to achieve immune balance and harmony: glycosyltransferase UGT76B1 inactivates *N*-hydroxy-pipecolic acid to suppress defense responses. The Plant Cell.

[85] (2007). Transgenic approaches for abiotic stress tolerance in plants: retrospect and prospects. Plant Cell Reports.

[86] (2016). Abscisic acid and abiotic stress tolerance in crop plants. Frontiers in Plant Science.

[87] (2012). A vacuolar β-glucosidase homolog that possesses glucose-conjugated abscisic acid hydrolyzing activity plays an important role in osmotic stress responses in *Arabidopsis*. The Plant Cell.

[88] (2000). Extracellular β-glucosidase activity in barley involved in the hydrolysis of ABA glucose conjugate in leaves. Journal of Experimental Botany.

[89] (2006). Activation of glucosidase via stress-induced polymerization rapidly increases active pools of abscisic acid. Cell.

[90] (2013). Abiotic stress responses in TEA [*Camellia sinensis* L (O) Kuntze]: an overview. Reviews in Agricultural Science.

[91] (2005). The use of abscisic acid analogues to analyse the substrate selectivity of UGT71B6, a UDP-glycosyltransferase of *Arabidopsis thaliana*. FEBS Letters.

[92] (1996). Resistance gene-dependent plant defense responses. The Plant Cell.

[93] (2021). Plant defense responses to biotic stress and its interplay with fluctuating dark/light conditions. Frontiers in Plant Science.

[94] (2008). Seed transmission of *Fusarium oxysporu*m f. sp. vasinfectum race 4 in California. The Journal of Cotton Science.

[95] (2002). Alternate energy-dependent pathways for the vacuolar uptake of glucose and glutathione conjugates. Plant Physiology.

[96] (2023). The function of UDP-glycosyltransferases in plants and their possible use in crop protection. Biotechnology Advances.

[97] (2020). Metabolomic and transcriptomic analyses of anthocyanin biosynthesis mechanisms in the color mutant *Ziziphus jujuba* cv. Tailihong. Journal of Agricultural and Food Chemistry.

[98] (2010). Cloning and heterologous expression of glycosyltransferases from *Malus* x *domestica* and *Pyrus communis*, which convert phloretin to phloretin 2'-O-glucoside (phloridzin). Plant Science.

