# Supplementary material for: Characterization of UGT71, a major glycosyltransferase family for triterpenoids, flavonoids and phytohormones-biosynthetic in plants
Source: For Res (Fayettev). 2024 Oct 31;4:e035. doi: 10.48130/forres-0024-0032 (PMC11564731; doi:10.48130/forres-0024-0032)
Supplement: Supplementary file 1 — Supplementary data to this article can be found online. [file FR-2024-4-0032-S1.zip › 10.48130_forres-0024-0032-Suppl-TableS1.pdf]

**Table S1.** The numbers of total UGTs found in different plants species that have been published in public databases.

| Number of UGTs | Species | Phylogenetic group(s) | | | | | | | | | | | | | | | | | | References |
|---|---|---|---|---|---|---|---|---|---|---|---|---|---|---|---|---|---|---|---|---|
| | | **A** | **B** | **C** | **D** | **F** | **E** | **G** | **H** | **I** | **G** | **K** | **L** | **M** | **N** | **O** | **P** | **Q** | **O** | |
| **1** | ***Chlamydomonas reinhaedtii*** | 0 | 0 | 0 | 0 | 0 | 0 | 0 | 0 | 0 | 0 | 0 | 0 | 0 | 0 | 0 | 0 | 0 | 1 | [97] |
| **2** | ***Chara braunii*** | 0 | 0 | 0 | 0 | 0 | 0 | 0 | 0 | 0 | 0 | 0 | 0 | 0 | 0 | 0 | 0 | 0 | 2 | [88] |
| **15** | ***Physcomitrella patens*** | 0 | 0 | 0 | 0 | 0 | 0 | 0 | 0 | 0 | 0 | 0 | 0 | 0 | 0 | 0 | 0 | 0 | 15 | [91] |
| **41** | ***Marchantia polymorpha*** | 0 | 0 | 0 | 0 | 0 | 0 | 0 | 0 | 0 | 0 | 0 | 0 | 0 | 0 | 0 | 0 | 0 | 41 | [101] |
| **56** | ***Carica papaya*** | 5 | 3 | 0 | 10 | 12 | 1 | 6 | 8 | 1 | 2 | 2 | 7 | 2 | 1 | 1 | 1 | 0 | 0 | [96] |
| **85** | ***Cucumis sativus*** | 13 | 1 | 2 | 12 | 12 | 2 | 11 | 5 | 0 | 2 | 1 | 16 | 2 | 1 | 3 | 5 | 0 | 0 | [86] |
| **100** | ***Dioscorea rotundata*** | 6 | 3 | 3 | 9 | 15 | 1 | 6 | 0 | 3 | 4 | 1 | 10 | 0 | 1 | 8 | 4 | 2 | 2 | [87] |
| **102** | ***Vitis vinifera*** | 23 | 1 | 0 | 3 | 14 | 6 | 5 | 2 | 6 | 4 | 2 | 9 | 1 | 1 | 0 | 3 | 3 | 0 | [105] |
| **117** | ***Ricinus communis*** | 11 | 4 | 2 | 17 | 8 | 3 | 15 | 4 | 8 | 2 | 1 | 17 | 3 | 1 | 2 | 1 | 2 | 12 | [97] |
| **123** | ***Arabidopsis thaliana*** | 14 | 4 | 4 | 13 | 25 | 4 | 7 | 9 | 1 | 2 | 2 | 18 | 1 | 1 | 0 | 0 | 0 | 6 | [111] |
| **129** | ***Ginkgo biloba*** | 17 | 3 | 0 | 0 | 9 | 1 | 4 | 0 | 0 | 2 | 14 | 12 | 9 | 0 | 6 | 0 | 1 | 7 | [108] |
| ***137*** | ***Vaccinium corymbosum*** | 20 | 1 | 3 | 10 | 16 | 3 | 24 | 1 | 1 | 3 | 1 | 19 | 3 | 0 | 8 | 11 | 2 | 9 | [94] |
| ***155*** | ***Mimulus guttatus*** | 24 | 2 | 3 | 11 | 19 | 2 | 12 | 1 | 4 | 2 | 15 | 18 | 1 | 1 | 10 | 3 | 1 | 2 | [1] |
| ***149*** | ***Morus notabilis*** | 17 | 4 | 2 | 14 | 30 | 3 | 8 | 5 | 2 | 3 | 7 | 16 | 5 | 1 | 7 | 3 | 1 | 17 | [24] |
| ***162*** | ***Solanum lycopersicum*** | 24 | 2 | 2 | 17 | 16 | 2 | 13 | 6 | 2 | 1 | 5 | 20 | 3 | 1 | 25 | 6 | 1 | 15 | [93] |
| ***181*** | ***Theobroma cacao*** | 17 | 5 | 1 | 21 | 22 | 5 | 9 | 9 | 8 | 6 | 2 | 24 | 6 | 1 | 2 | 11 | 2 | 20 | [112] |
| ***182*** | ***Linum usitatissimum*** | 16 | 5 | 6 | 25 | 24 | 1 | 21 | 8 | 9 | 4 | 5 | 24 | 3 | 1 | 0 | 4 | 2 | 19 | [102] |
| ***184*** | ***Oryza sativa*** | 15 | 4 | 7 | 20 | 36 | 2 | 19 | 7 | 6 | 3 | 1 | 21 | 8 | 2 | 6 | 6 | 0 | 16 | [95] |
| ***187*** | ***Medicago truncatula*** | 30 | 4 | 0 | 55 | 58 | 2 | 39 | 4 | 5 | 9 | 0 | 33 | 2 | 1 | 3 | 3 | 1 | 20 | [90] |
| ***234*** | ***Populus trichocarpa*** | 14 | 2 | 6 | 15 | 53 | 8 | 36 | 6 | 5 | 7 | 2 | 23 | 8 | 1 | 3 | 3 | 3 | 0 | [110] |
| ***294*** | ***Ziziphus jujuba*** | 55 | 9 | 3 | 41 | 31 | 4 | 34 | 6 | 3 | 8 | 7 | 30 | 2 | 1 | 18 | 13 | 4 | 21 | [107] |
| ***379*** | ***Eucalyptus grandis*** | 30 | 6 | 6 | 35 | 74 | 2 | 44 | 9 | 9 | 20 | 2 | 47 | 6 | 3 | 11 | 4 | 4 | 28 | [89] |
