# Supplementary figures and images for: Characterization of UGT71, a major glycosyltransferase family for triterpenoids, flavonoids and phytohormones-biosynthetic in plants
Source: For Res (Fayettev). 2024 Oct 31;4:e035. doi: 10.48130/forres-0024-0032 (PMC11564731; doi:10.48130/forres-0024-0032)

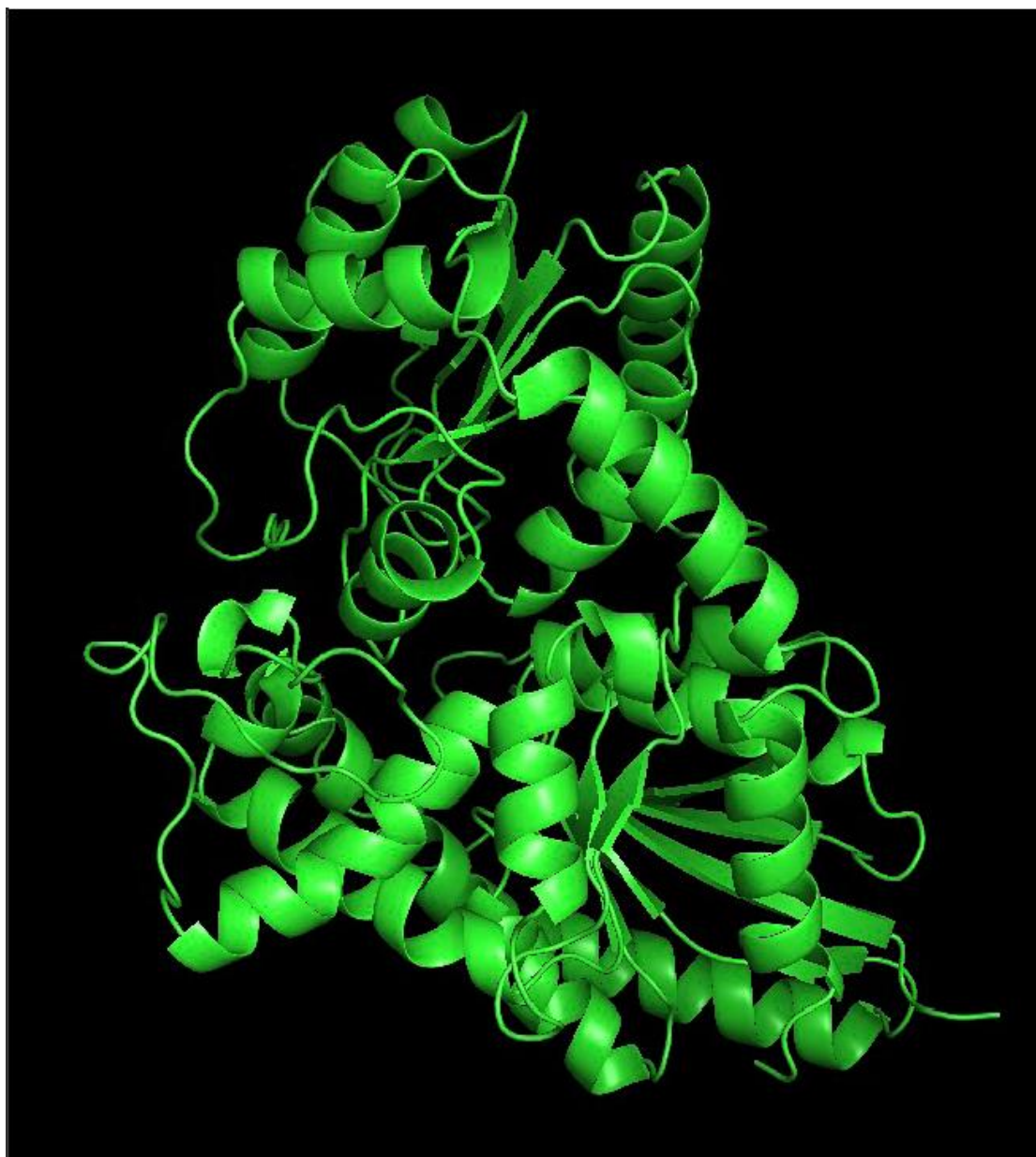

**Supplementary Fig. S1.** Modeled 3D structure of UGT71C3.

Supplement: Supplementary file 1 — Supplementary data to this article can be found online. [file FR-2024-4-0032-S1.zip › 10.48130_forres-0024-0032-Suppl-FigureS1.pdf]
